# Laparoscopic anatomical liver resection versus enucleation in the treatment of giant hepatic hemangioma larger than 10 cm: a retrospective study

**DOI:** 10.1186/s12893-026-03551-6

**Published:** 2026-02-27

**Authors:** Hongjun Huang, Minjie Lin, Zhiqiang Huang, Zhiming Wu, Jiangtao Li

**Affiliations:** 1 Department of Hepatobiliary Pancreatic Surgery, The Second Affiliated, Hospital Zhejiang University School of Medicine, Hangzhou, 310009 China; 2 Department of General Surgery, Shaoxing Central Hospital, Central Hospital affiliated to Shaoxing University, Shaoxing, 312030 China

**Keywords:** Laparoscopy, Giant hepatic hemangioma, Anatomical liver resection, Enucleation

## Abstract

**Background:**

Hepatic hemangiomas (HH) are the most common benign liver tumors. This study aimed to provide a clinical basis for selecting the most appropriate surgical approach for managing giant hepatic hemangiomas (> 10 cm).

**Methods:**

We retrospectively analyzed clinical data from 126 patients with giant hepatic hemangiomas treated at our hospital between January 2019 and May 2025. Of these, 54 had right hemi-hepatic hemangiomas, and 72 had left hemi-hepatic hemangiomas. Patients were divided into laparoscopic anatomical liver resection (LR) and laparoscopic hemangioma enucleation (LE) groups based on surgical approaches. Intraoperative parameters, postoperative outcomes, length of hospital stay, and complication rates were compared between the two groups.

**Results:**

No cases required conversion to open surgery. In patients with right hemi-hepatic hemangiomas, LE showed significantly shorter operative time [230.0 (175.0, 280.0) vs. 355.0 (235.0, 555.0) min, *P* = 0.001], shorter occlusion time [30.0 (15.0, 45.0) vs. 55.0 (45.0, 77.5) min, *P* < 0.05], less blood loss [200 vs. 300 mL, *P* < 0.001], and fewer autologous blood transfusions [12 vs. 15, *P* < 0.05] compared to LR. In contrast, no significant differences were observed in operative or postoperative outcomes between LR and LE for left hemi-hepatic hemangiomas (*P* > 0.05). Overall, across the entire cohort, there were no significant differences in operative time, occlusion time, hospital stay, or complication rates between LR and LE groups.

**Conclusion:**

Both LR and LE are safe and effective for treating giant hepatic hemangiomas. However, LE is particularly recommended for right hemi-hepatic hemangiomas, due to shorter operative and occlusion times, reduced intraoperative blood loss, and greater preservation of normal liver tissue.

## Background

Hepatic hemangiomas (HH) are the most common benign liver tumors, with a prevalence of approximately 4–20% in the general population [1]. They are more frequently discovered in adults aged 30–50 years, with a female-to-male ratio of approximately 4.5:1 to 5:1 [2]. Most HHs are asymptomatic and require no intervention. However, giant hepatic hemangiomas, defined as tumors exceeding 10 cm in diameter, may compress adjacent organs or vessels (e.g., gastric outlet, hepatic vein, portal vein, hepatic artery, or inferior vena cava), thereby increasing surgical complexity and risk.

Advancements in laparoscopic techniques have made laparoscopic resection the preferred approach for giant HHs, offering benefits such as reduced trauma, faster recovery, and improved cosmetic outcomes [3, 4], However, the optimal resection method remains debated. Some studies advocate for laparoscopic anatomical liver resection (LR) to control intraoperative bleeding, particularly for large or complexly located tumors, as it involves resecting the entire liver segment containing the tumor [5–8]. Conversely, others recommend laparoscopic hemangioma enucleation (LE), which removes the tumor along the anatomical plane between the tumor and normal liver tissue. LE is generally considered simpler, faster, and better at preserving liver function, especially for tumors located at the liver’s edge [9–11]. Based on the experience of laparotomy, enucleation is superior to liver resection, with advantages such as less bleeding, shorter operation time, and fewer complications [12]. Tumor size and location are critical factors influencing the feasibility of laparoscopic approaches and intraoperative bleeding risk, yet no consensus has been reached regarding the optimal approach based on these factors [13–16].

This study retrospectively analyzes data from patients treated for giant hepatic hemangiomas (> 10 cm) at a single center. We compared the outcomes of LR and LE for giant HHs across the whole liver and in different hemi-livers, with the aim of providing a clinical basis for selecting the most appropriate surgical approach, particularly in terms of operative time and intraoperative blood loss, followed by complications, drainage tube removal time, and length of hospital stay.

## Methods

### Patient selection

**Fig. 1:**
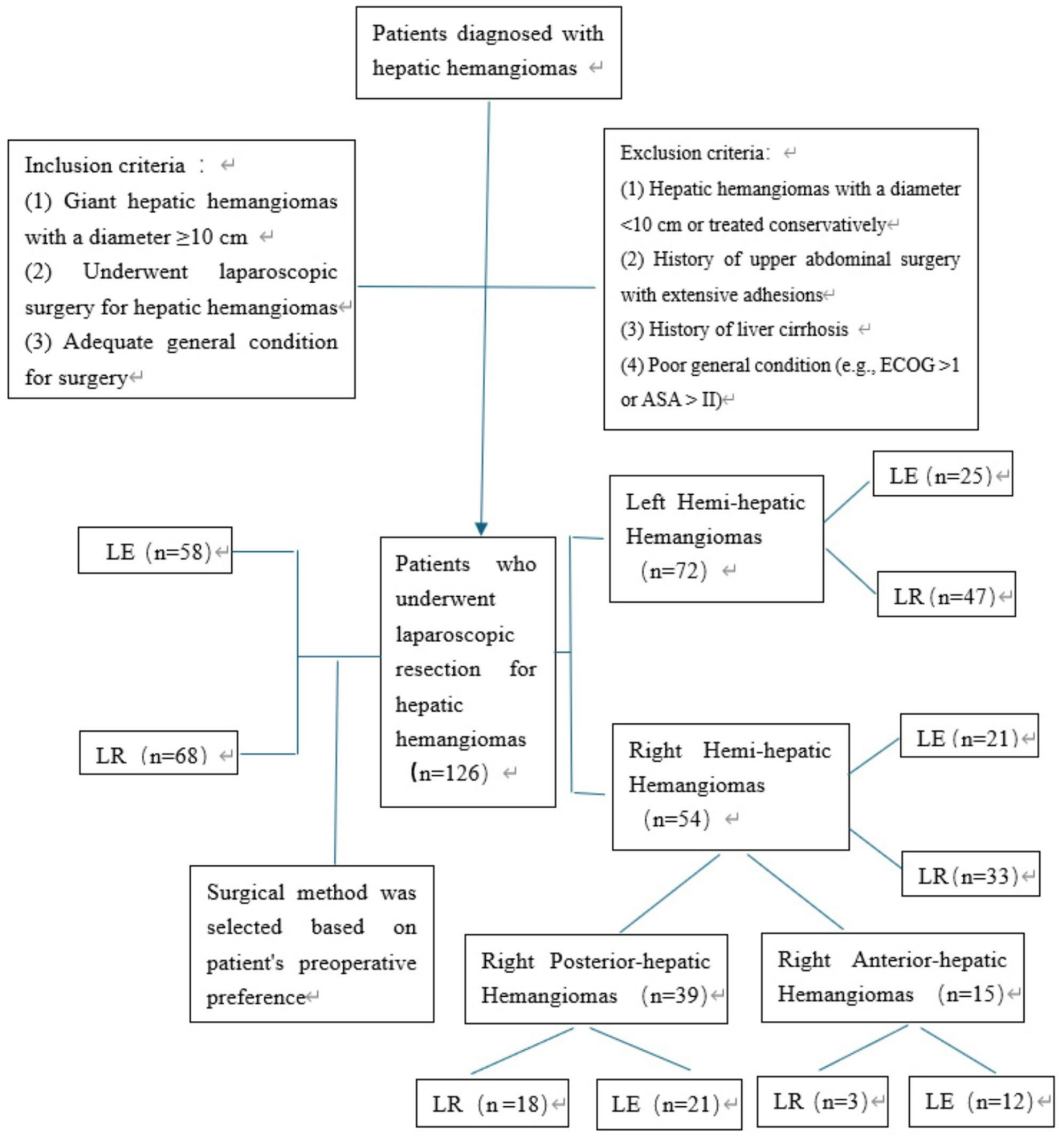
Flowchart depicting inclusion and exclusion criteria for patients with hepatic hemangiomas and subsequent classification by surgical approach: laparoscopic hemangioma enucleation (LE) or laparoscopic anatomic liver resection (LR)

The study reviewed the data of 126 patients who had laparoscopic hepatic hemangioma removal at our institution between January 2019 and May 2025 (Fig. 1). The study protocol was reviewed and approved by the Ethics Committee of Shaoxing Central Hospital (Approval No. 2025-14-001). All patients had previously provided written informed consent for their surgical procedures and for the potential secondary use of their anonymized clinical data for research purposes, in line with institutional policy. The inclusion criteria were as follows: (1) Giant hepatic hemangioma with a diameter of ≥ 10 cm; (2) Underwent laparoscopic surgery for hepatic hemangioma; and(3) Acceptable general condition with no severe comorbidities affecting major organ systems (including heart, lung, liver, kidney, or coagulation function) that would contraindicate surgery.; the exclusion criteria were as follows: (1) Hemangioma diameter < 10 cm or conservative treatment; (2) Prior upper abdominal surgery resulting in significant adhesions; (3) Evidence of liver cirrhosis on imaging or clinical evaluation; (4) Comorbidities with other medical conditions, such as kidney disease, severe diabetes, etc., which are detrimental to postoperative recovery diseases that affect; The LR approach is defined as the removal of liver segments containing HH lesions, regardless of the tumor capsule’s border. The LE procedure is defined as the removal of HH along the tumor capsule, without losing normal tissue. The choice between LE and LR was made according to the patient’s preoperative preference after detailed discussion with the surgical team regarding the risks and benefits of each approach. Decision-making as follows: the surgeon first evaluated tumor location, size, proximity to major vessels (Glissonean pedicles, hepatic veins, IVC), and technical feasibility of a safe enucleation plane. After standardized counseling by the same senior surgeon, including the advantages (parenchyma-sparing) and disadvantages (risk of bleeding or incomplete removal) of LE, versus the advantages (clear margins) and disadvantages (greater parenchymal loss) of LR, and the final decision was made according to the patient’s informed preference.

### Surgical procedures and perioperative management

All procedures were performed by the same experienced chief surgeon using the conventional five-trocar laparoscopic technique. Routine preoperative blood preparation and readiness for autologous blood transfusion were ensured for all patients. Patient positioning was adjusted intraoperatively as required. Under general anesthesia, patients were placed in the supine position. A 10-mm supra-umbilical trocar served as the observation port; for right-lobe tumors, this port could be shifted to the right epigastric region based on the patient’s body habitus (height and weight). Carbon dioxide pneumoperitoneum was maintained at 13–15 mmHg (1 mmHg = 0.133 kPa).

After laparoscopic exploration of the abdominal cavity to confirm the location and extent of the hemangioma, the remaining four working trocars were placed in a “V” configuration, with the tumor positioned at the apex of the V to optimize visualization and maneuverability. The hepatic hilum was identified, the perihepatic ligament was released to fully expose the tumor. Subsequently, either laparoscopic hemangioma enucleation (LE) or laparoscopic anatomic liver resection (LR) was carried out according to the preoperative plan.

### Laparoscopic hemangioma enucleation (LE)

**Fig. 2:**
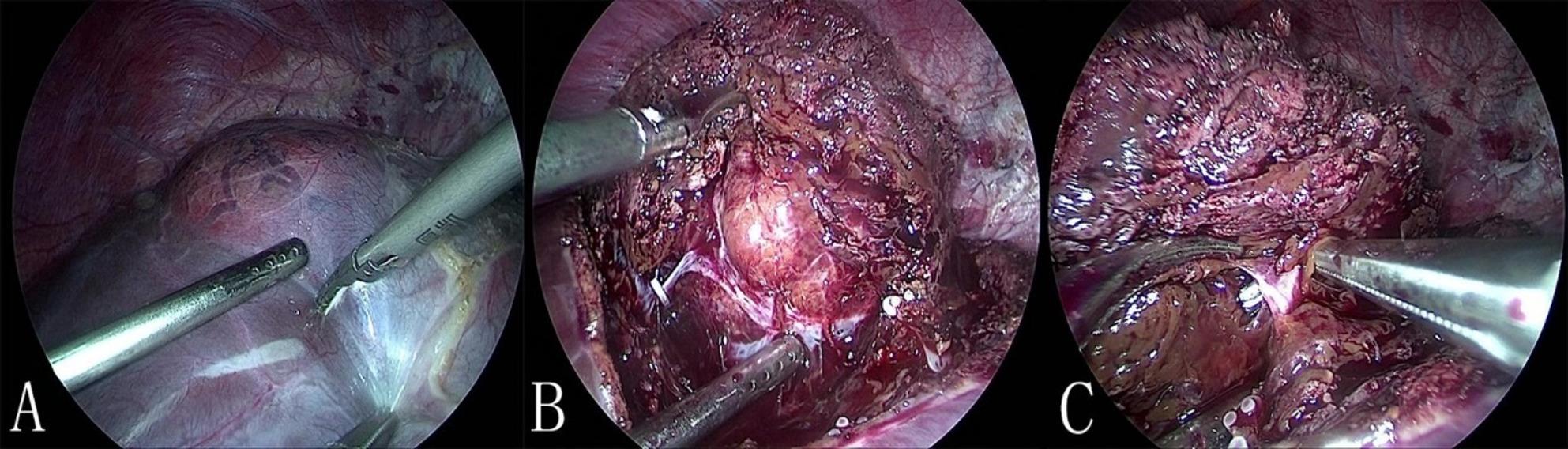
Laparoscopic enucleation of hepatic hemangioma. **A** Separation began from the easy entry site. **B** A tumor’s capsule was elevated, dissecting along peritumoral plane. **C** Vessels and ducts were controlled with clips, electrocautery, and sutures.

After the pringle maneuver was performed, the appropriate entry point was selected according to the “Easy-First” principle. Dissection was initiated at this readily accessible site (Fig. 2A), then part of the wall of the hemangioma is elevated (Fig. 2B) and the tumor is progressively separated along the peritumoral plane by harmonic scalpel. As bile ducts or vessels were encountered during dissection, they were identified, secured (with clips or ligation), and divided. Hemostasis was achieved using a combination of titanium clips, electrocautery, and absorbable sutures as needed (Fig. 2C).

### Laparoscopic anatomical liver resection (LR)

The corresponding segmental or sectional branch of the Glissonean pedicle was first isolated and divided at the hepatic hilum. Liver parenchymal transection was then performed along the demarcated intersegmental plane. The liver parenchyma was dissected with a harmonic scalpel, Hepatic vein branches or bile ducts were ligated and divided, with hemostasis achieved using Hem-o-lock (Johnson & Johnson) or absorbable clips, bipolar electrocoagulation, or sutures as needed. The specimen was removed by extending the observation port, and a hepatic drainage tube was routinely placed after ensuring adequate hemostasis.

### Postoperative management and follow-up protocol

All patients received intravenous prophylactic antibiotics for 24–48 h postoperatively to reduce the risk of surgical site or intra-abdominal infection. Hepatoprotective agents, hemostatic medications, and rehydration therapy were routinely administered. On the third postoperative day, a comprehensive laboratory evaluation was conducted, including blood tests, liver and kidney function, coagulation parameters and C reactive protein (CRP). All patients in this cohort were routinely followed up at our institution. Surveillance imaging—consisting of abdominal ultrasound and/or contrast-enhanced CT or MRI—was scheduled at 3, 6, 12, and 36 months postoperatively to assess for recurrence, residual lesions, or other complications.

### Outcome measures

The following outcomes were recorded: rate of conversion to laparotomy, operative time, intraoperative blood loss, hepatic hilum occlusion time, intraoperative blood transfusion, postoperative length of stay (POLS), drainage tube removal time, and postoperative day 3 assessments of blood count, coagulation function, and liver function levels; Complications, including postoperative bleeding, bile leakage, abdominal infection, and incision infection, and other complications were monitored.

### Statistical analysis

Statistical analyses were performed using SPSS version 27.0 (IBM Corp, Armonk, NY, USA). The Shapiro–Wilk test was used to assess normality of continuous variables. Categorical variables are presented as frequencies and percentages and were compared using the *χ*² test or *Fisher*’s exact test, as appropriate. Continuous variables are expressed as mean ± standard deviation (SD) when normally distributed or as median (interquartile range, IQR: *P25*,* P75*) when non-normally distributed; comparisons were performed using the independent-samples t-test or the Mann–Whitney U test, *P* < 0.05 was considered statistically significant.

## Results

### Overall efficacy comparison between LR and LE groups

#### Patient characteristics

Between January 2019 and May 2025, a total of 126 patients with giant hepatic hemangiomas (HHs) underwent laparoscopic surgery at our institution. The cohort included 38 males and 88 females, with a median age of 52 years. Patients were divided into two groups: 68 underwent LR, and 58 underwent LE. There were no cases in which objective intraoperative feasibility thresholds (e.g., proximity to major vessels or inability to identify a safe enucleation plane) led to a surgeon-directed recommendation overriding patient preference. Baseline characteristics, including age, sex, body mass index (BMI), American Society of Anesthesiologists (ASA) classification, tumor size, and preoperative laboratory tests (e.g., ALT, AST, TBIL, PT, ALB), showed no statistically significant differences between groups (*P* > 0.05, Table 1), ensuring comparability.

**Table 1:** Comparison of baseline characteristics between LR and LE groups

Variable	LR (*n* = 68)	LE (*n* = 58)	*P* value
Age(years)	51.39 ± 9.3	51.8 ± 8.6	0.398^#^
Male/female	22/46	16/42	0.697^*^
BMI	23.3 ± 1.8	23.5 ± 1.4	0.493^#^
ASA (*n*)			
I	50	44	
II	18	14	0.839^*^
Tumor size (cm)	15.0 (13.25, 16.0)	15.0 (12.0, 16.0)	0.584^Ÿ^
Preoperative ALT (U/L)	18.0 (13.0, 26.0)	24.0 (15.0, 31.5)	0.067^Ÿ^
Preoperative AST (U/L)	19.0 (16.0, 25.8)	23.0 (17.0, 29.0)	0.077^Ÿ^
Preoperative TBIL (umol/L)	11.7 (9.2, 14.7)	11.5(9.7, 19.3)	0.793^Ÿ^
Preoperative PT (s)	11.5 (10.6, 12.2)	11.5 (8.5, 18.0)	0.893^Ÿ^
Preoperative PLT	187.4 ± 23.4	190.2 ± 22.1	0.493^#^
Preoperative ALB (g/L)	40.5 (37.8, 43.7)	42.3 (39.4, 43.8)	0.075^Ÿ^

Data are presented as mean ± SD for normally distributed continuous variables (^#^), as median (IQR) for non-normally distributed continuous variables (^Ÿ^), and as *n* (%) for categorical variables (^*^). *BMI* Body Mass Index, *ASA* American Society of Anesthesiologists, *ALT* Alanine Transaminase, *AST* Aspartate Aminotransferase, *TBIL* Total Bilirubin, *PT* Prothrombin Time, *PLT* Platelet, *ALB* Albumin, *HGB* Hemoglobin, *POLS* Postoperative Length of Stay

#### Operative outcomes

All procedures were successfully completed, with no conversions to open surgery. Hemoglobin (HGB) levels were higher in the LR group [119.5 (109.5, 126.0) vs. 106.0 (100.0, 116.5) g/L, *P* = 0.001]. The LR group also had a shorter time to drainage tube removal [4.5 (4.0, 5.0) vs. 6.0 (4.0, 6.0) days, *P* = 0.044]. No significant differences were observed in operative time, occlusion time, blood loss, transfusion rates, postoperative liver function tests (TBIL, ALB, PT), or POLS between groups (*P* > 0.05). Details were shown in Table 2.

**Table 2:** Comparison of operative outcomes between LR and LE groups

Variable	LR (*n* = 68)	LE (*n* = 58)	*P* value
Operation time (min)	150.0 (110.0, 242.5)	180.0 (134.8, 240.0)	0.381^Ÿ^
Occlusion time (min)	16.0 (12.0, 43.8)	18.0 (10.0, 45.0)	0.860^Ÿ^
Blood loss (ml)	290.0 (50.0, 570.0)	110.0 (66.8, 602.5)	0.862^Ÿ^
Transfusion (*n*)			
autologous blood transfusion	18 (26.5%)	13 (22.4%)	0.680^*^
allogeneic blood transfusion	15 (22.1%)	13 (22.4%)	1^*^
Conversion (*n*)	0	0	
Postoperative PT (s)	11.2 (10.3, 12.9)	11.8 (10.5, 12.9)	0.850^Ÿ^
Postoperative TBIL (umol/L)	14.4 (11.1, 17.7)	15.9 (9.3, 18.6)	0.824^Ÿ^
Postoperative ALB (g/L)	32.7 (30.4, 35.5)	31.4 (30.0, 34.0)	0.133^Ÿ^
Postoperative HGB (g/L)	119.5 (109.5, 126.0)	106.0 (100.0, 116.5)	0.001^Ÿ^
Remove drainage tube (days)	4.5 (4.0, 5.0)	6.0 (4.0, 6.0)	0.044^Ÿ^
POLS (days)	11.4 ± 3.7	11.9 ± 3.8	0.977^#^

Data are presented as mean ± SD for normally distributed continuous variables (^#^), as median (IQR) for non-normally distributed continuous variables (^Ÿ^), and as *n* (%) for categorical variables (^*^). *BMI* Body Mass Index, *ASA* American Society of Anesthesiologists, *ALT* Alanine Transaminase, *AST* Aspartate Aminotransferase, *TBIL* Total Bilirubin, *PT* Prothrombin Time, *PLT* Platelet, *ALB* Albumin, *HGB* Hemoglobin, *POLS* Postoperative Length of Stay

#### Postoperative complications

Complications were minimal and comparable between groups. Bile leakage occurred in three cases (one in LR, two in LE, *P* = 0.594), with associated infections in each case. Pleural effusion was observed in six cases (three in each group, *P* = 1.000). No cases of postoperative hemorrhage were reported. No statistically significant differences in complication rates were observed (*P* > 0.05). Details were shown in Table 3.

**Table 3:** Comparison of postoperative complications between LR and LE groups

Complications (*n*)	LR (*n* = 68)	LE (*n* = 58)	*P* value
Bile leakage	1 (1.5%)	2 (3.4%)	0.594^*^
Pleura effusion	3 (4.4%)	3 (5.2%)	1^*^
Infection	1 (1.5%)	2 (3.4%)	0.594^*^
Hemorrhage	0	0	

Data are presented as *n* (%) for categorical variables (^*^)

#### Long-term outcomes

The median follow-up time was 24 months (range 3–36 months) for the entire cohort. No residual hemangioma, incomplete resection, or recurrence was observed in either the LR or LE group during follow-up. No patient reported delayed symptoms (abdominal pain, fullness, or jaundice) attributable to remnant or recurrent hemangioma.

### Subgroup analysis: right Hemi-hepatic hemangiomas

Of the 54 patients with right hemi-hepatic hemangiomas, 21 underwent LR, and 33 underwent LE. Baseline characteristics showed no significant differences between groups (*P* > 0.05). LE demonstrated significant advantages over LR, including shorter operative time [230.0 (175.0, 280.0) vs. 355.0 (235.0, 555.0) min, *P* = 0.001], shorter occlusion time [30.0 (15.0, 45.0) vs. 55.0 (45.0, 77.5) min, *P* = 0.001], less blood loss [500.0 (110.0, 995.0) vs. 800.0 (405.0, 2495.0) mL, *P* = 0.011], and fewer autologous blood transfusions [12 vs. 15, *P* = 0.024]. No significant differences were observed in other variables, including allogeneic transfusion, postoperative PT, TBIL, ALB, HGB, drainage tube removal time, POLS, or complications (*P* > 0.05). Details were shown in Table 4. Additionally, there are 39 cases in the right posterior lobe, 18 underwent LR, and 21 underwent LE. Intraoperative outcomes favored LE. Operative time was significantly shorter in the LE group [367.5 (220.0, 570.0) vs. 250.0 (175.0, 290.0) min, *P* = 0.008], and occlusion time was also reduced [60.0 (43.8, 82.5) vs. 45.0 (20.0, 47.0) min, *P* = 0.004]. Blood loss was lower in the LE group [1150.0 (397.5, 2503.0) vs. 600.0 (305.0, 1495.0) mL, *P* = 0.099], though the difference was not statistically significant. No significant differences were observed in other variables, including postoperative outcomes. Details were shown in Table 5. In right anterior lobe, the LR group had only 3 cases, while the LE group had 12. Due to the small sample size and low statistical power, which may not reflect the true situation, no statistical analysis was performed.

**Table 4:** Comparison of characteristics and outcomes for right Hemi- hepatic hemangiomas

Variable	LR (*n* = 21)	LE (*n* = 33)	*P* value
Age (years)	46.4 ± 8.1	50.3 ± 9.5	0.212^#^
Male/female	7/14	9/24	0.762^*^
BMI	23.2 ± 1.2	23.6 ± 1.4	0.285^#^
ASA (*n*)			
I	17	25	
II	4	8	0.747^*^
Tumor size(cm)	16.0 ± 1.6	14.5 ± 2.8	0.125^#^
Preoperative ALT(U/L)	16.0 (15.0, 17.0)	24.0 (13.0, 28.0)	0.295^Ÿ^
Preoperative AST(U/L)	19.0 (17.0, 24.5)	22.0 (15.0, 30.0)	0.374^Ÿ^
Preoperative TBIL (umol/L)	14.2 (10.0, 15.9)	10.6 (8.3, 17.8)	0.211^Ÿ^
Preoperative PT(s)	11.3 (10.5, 11.8)	11.1 (9.9, 12.1)	0.295^Ÿ^
Preoperative PLT	183.4 ± 19.4	186.2 ± 20.2	0.616^#^
Preoperative ALB(g/L)	42.9 (37.1, 47.6)	43.0 (41.9, 45.5)	0.683^Ÿ^
Operation time (min)	355.0 (235.0, 555.0)	230.0 (175.0, 280.0)	0.001^Ÿ^
Occlusion time (min)	55.0 (45.0, 77.5)	30.0 (15.0, 45.0)	0.001^Ÿ^
Blood loss (ml)	800.0 (405.0, 2495.0)	500.0 (110.0, 995.0)	0.011^Ÿ^
Blood transfusion (n)			
autologous blood transfusion	15	12	0.024^*^
allogeneic blood transfusion	10	9	0.150^*^
Conversion (*n*)	0	0	
Postoperative PT(s)	11.3 (10.2, 14.7)	11.0 (9.9, 12.3)	0.136^Ÿ^
Postoperative TBIL (umol/L)	14.5 (11.5, 24.8)	15.9 (7.8, 18.7)	0.234^Ÿ^
Postoperative ALB (g/L)	29.0 (26.5, 31.8)	30.5 (29.0, 33.8)	0.141^Ÿ^
Postoperative HGB (g/L)	113.0 ± 14.4	113.3 ± 7.3	0.061^#^
Remove drainage tube (days)	7.0 (4.0, 8.0)	6.0 (5.0, 7.0)	0.370^Ÿ^
POLS (days)	14.2 ± 3.9	12.6 ± 4.3	0.872^#^
Complications (*n*)			
Bile leakage	1	2	1^*^
Pleura effusion	3	3	0.667^*^
Infection	1	2	1^*^
Hemorrhage	0	0	

Data are presented as mean ± SD for normally distributed continuous variables (^#^), as median (IQR) for non-normally distributed continuous variables (^Ÿ^), and as *n* (%) for categorical variables (^*^). *BMI* Body Mass Index, *ASA* American Society of Anesthesiologists, *ALT* Alanine Transaminase, *AST* Aspartate Aminotransferase, *TBIL* Total Bilirubin, *PT* Prothrombin Time, *PLT* Platelet, *ALB* Albumin, *HGB* Hemoglobin, *POLS* Postoperative Length of Stay

**Table 5:** Comparison of characteristics and outcomes for right Posterior- hepatic hemangiomas

Variable	LR (*n* = 18)	LE (*n* = 21)	*P* value
Age (years)	47.3 ± 8.4	49.8 ± 8.4	0.495^#^
Male/female	6/12	3/18	0.255^*^
BMI	23.2 ± 1.3	23.3 ± 1.6	0.833^#^
ASA (*n*)			
I	15	18	
II	3	3	1^*^
Tumor size(cm)	16.1 ± 1.6	15.0 ± 1.5	0.604^#^
Preoperative ALT(U/L)	19.5 (12.0, 40.5)	21.0 (12.5, 27.0)	0.994^Ÿ^
Preoperative AST(U/L)	22.9 ± 5.3	22.3 ± 7.6	0.071^#^
Preoperative TBIL (umol/L)	14.7 (9.7, 16.1)	10.6 (8.9, 20.3)	0.398^Ÿ^
Preoperative PT(s)	11.5 ± 0.8	12.0 ± 0.9	0.292^#^
Preoperative PLT	180.4 ± 29.4	183.2 ± 20.2	0.727^#^
Preoperative ALB(g/L)	40.3 (37.0, 47.7)	42.6 (41.9, 43.6)	0.464^Ÿ^
Operation time (min)	367.5 (220.0, 570.0)	250.0 (175.0, 290.0)	0.008^Ÿ^
Occlusion time (min)	60.0 (43.8, 82.5)	45.0 (20.0, 47.0)	0.004^Ÿ^
Blood loss (ml)	1150.0 (397.5, 2503.0)	600.0 (305.0, 1495.0)	0.099^Ÿ^
Blood transfusion (*n*)			
autologous blood transfusion	12	9	0.200^*^
allogeneic blood transfusion	10	7	0.206^*^
Conversion (*n*)	0	0	
Postoperative PT(s)	12.1 ± 2.1	11.3 ± 1.6	0.093^#^
Postoperative TBIL (umol/L)	18.0 ± 7.4	15.5 ± 8.1	0.930^#^
Postoperative ALB (g/L)	29.0 ± 2.8	30.14 ± 2.35	0.160^#^
Postoperative HGB (g/L)	119.0 (112.8, 125.3)	113.0 (97.0, 118.5)	0.051^Ÿ^
Remove drainage tube (days)	6.0 (4.0, 7.0)	6.0 (5.0, 7.0)	0.897^Ÿ^
POLS (days)	13.0 (10.0, 16.3)	12.0 (10.0, 13.5)	0.350^Ÿ^
Complications (*n*)			
Bile leakage	1	1	1^*^
Pleura effusion	3	3	1^*^
Infection	1	2	1^*^
Hemorrhage	0	0	

Data are presented as mean ± SD for normally distributed continuous variables (^#^), as median (IQR) for non-normally distributed continuous variables (^Ÿ^), and as *n* (%) for categorical variables (^*^). *BMI* Body Mass Index, *ASA* American Society of Anesthesiologists, *ALT* Alanine Transaminase, *AST* Aspartate Aminotransferase, *TBIL* Total Bilirubin, *PT* Prothrombin Time, *PLT* Platelet, *ALB* Albumin, *HGB* Hemoglobin, *POLS* Postoperative Length of Stay

### Subgroup analysis: left hemi-hepatic hemangiomas

### Patient characteristics and operative outcomes

Of the 72 patients with left hemi-liver hemangiomas, 47 underwent LR, and 25 underwent LE. Baseline characteristics were comparable between groups (*P* > 0.05). No significant differences were observed in operative time, occlusion time, blood loss, transfusion rates, postoperative liver function tests, HGB, drainage tube removal time, POLS, or complications (*P* > 0.05). Notably, no cases of bile leakage, pleural effusion, infection, or hemorrhage were reported in either group. Details were shown in Table 6.

**Table 6:** Comparison of characteristics and outcomes for left Hemi-Hepatic hemangiomas

Variable	LR (*n* = 47)	LE (*n* = 25)	*P* value
Age(years)	53.6 ± 9.0	53.7 ± 6.9	0.058^#^
Male/female (*n*)	15/32	7/18	0.794^*^
BMI	23.2 ± 1.9	23.9 ± 1.5	0.115^#^
ASA (*n*)			
I	33	19	
II	14	6	0.783^*^
Tumor size(cm)	14.4 ± 2.4.	14.8 ± 3.2	0.071^#^
Preoperative ALT (U/L)	18.0 (13.0, 26.0)	26.0 (17.0, 34.0)	0.082^Ÿ^
Preoperative AST (U/L)	19.0 (16.0, 30.0)	24.0 (19.0, 27.0)	0.074^Ÿ^
Preoperative TBIL (umol/L)	11.1 (8.7, 13.6)	11.5 (9.7, 19.3)	0.230^Ÿ^
Preoperative PT (s)	11.5 (10.6, 12.6)	12.0 (11.4, 12.4)	0.133^Ÿ^
Preoperative PLT	189.8 ± 18.4	192.2 ± 22.4	0.627^#^
Preoperative ALB (g/L)	40.3 ± 3.2	40.3 ± 2.5	0.358^#^
Operation time (min)	132.2 ± 45.0	137.0 ± 39.2	0.335^#^
Occlusion time (min)	14 (10.0, 17.0)	15.0 (9.5, 18.0)	0.854^Ÿ^
Blood loss (ml)	100.0 (40.0, 310.0)	50.0 (49.0, 105.0)	0.195^Ÿ^
Transfusion (*n*)			
autologous blood transfusion.	5	4	0.709^*^
allogeneic blood transfusion	3	1	1^*^
Conversion (*n*)	0	0	
Postoperative PT (s)	11.0 (10.7, 12.7)	12.0 (11.7, 13.0)	0.116^Ÿ^
Postoperative TBIL (*u*mol/L)	14.2 (10.9, 16.1)	15.6 (10.0, 18.6)	0.493^Ÿ^
Postoperative ALB (g/L)	34.4 (32.3, 36.1)	33.0 (31.3, 34.5)	0.203^Ÿ^
Postoperative HGB (g/L)	118.3 ± 14.7	111.8 ± 21.9	0.138^#^
Remove drainage tube	4.0 (4.0, 5.0)	4.0 (4.0, 5.5)	0.333^Ÿ^
POLS (days)	10.1 ± 2.9	11.1 ± 3.2	0.290^#^
Complications (*n*)			
Bile leakage	0	0	
Pleura effusion	0	0	
Infection	0	0	
Hemorrhage	0	0	

Data are presented as mean ± SD for normally distributed continuous variables (^#^), as median (IQR) for non-normally distributed continuous variables (^Ÿ^), and as *n* (%) for categorical variables (^*^). *BMI* Body Mass Index, *ASA* American Society of Anesthesiologists, *ALT* Alanine Transaminase, *AST* Aspartate Aminotransferase, *TBIL* Total Bilirubin, *PT* Prothrombin Time, *PLT* Platelet, *ALB* Albumin, *HGB* Hemoglobin, *POLS* Postoperative Length of Stay

## Discussion

Our results are in line with the broader experience of minimally invasive liver surgery, where laparoscopic liver resection has been shown to provide perioperative benefits without compromising short-term outcomes when compared with open approaches. In a recent meta-analysis including patients undergoing resection of large hepatocellular carcinomas, laparoscopic liver resection was associated with reduced blood loss, lower complication rates and shorter hospital stay despite comparable tumor size and complexity relative to open surgery [17]. Although our study focuses on benign giant hemangiomas, these data reinforce that laparoscopic approach remains safe and effective for large, technically challenging liver lesions when performed in high-volume, experienced centers. In this study, we compared the efficacy of LR and LE for giant hepatic hemangiomas (> 10 cm) across the entire liver, as well as stratified by right and left hemi-hepatic locations. Both techniques proved safe and effective overall, with no significant differences in key surgical outcomes, including operative time, occlusion time, blood loss, and hospital stay. Notably, LR group showed minor advantages in postoperative recovery, evidenced by higher hemoglobin levels, and earlier drainage tube removal. For HHs in the right liver, LE group demonstrated significant advantages over LR group, including shorter operative and occlusion times, reduced intraoperative blood loss, indicating less surgical trauma and liver damage. No significant differences were observed in other parameters, such as blood transfusion rates or hospital stay. In contrast, for left hemi-liver hemangiomas, no significant differences were found between LR and LE group in surgical or postoperative outcomes, except that LE group better preserved normal liver tissue.

The exact indications for hepatic hemangioma surgery are not yet been standardized and are summarized in the literature as follows [11, 18]: (1) Tumor diameter > 10 cm [19]. (2) Symptoms including fever [6], compression of peripheral organs (e.g. epigastric discomfort, abdominal distension, nausea and vomiting [7]). (3) Rapid growth (> 2 cm/year). (4) Thrombocytopenia, such as Kasabach-Merritt syndrome (KMS). (5) Atypical, difficult to differentiate from other liver tumors. (6) Anxiety about disease, affecting quality of life despite psychological counseling. In this study, all 126 cases involved giant hemangiomas (> 10 cm). Indications for surgery were distributed as follows: 40 patients were asymptomatic (surgery driven by size alone), 24 presented with compressive symptoms, 20 exhibited rapid tumor growth, and 42 underwent resection primarily due to persistent anxiety affecting quality of life after counseling. None of our patients exhibited KMS or related abnormalities in coagulation/fibrinolysis, possibly due to the low reported incidence of Kasabach-Merritt syndrome (KMS) in adult patients with giant hepatic hemangiomas [20, 21].

For hepatic hemangiomas, laparoscopic resection [4, 14, 19, 22, 23] is the preferred treatment, outperforming other methods like radiofrequency ablation [24] or hepatic artery embolization [25], which is thorough, non-recurrent, and safe. Nevertheless, the optimal choice between LR and LE for giant hemangiomas remains controversial. Open surgery data suggest that enucleation offers advantages over resection, including less bleeding, shorter operative times, and fewer complications; Zhang et al. [26]found no difference in operation time, blood loss, complications or hospital stay between enucleation and resection for hemangiomas > 10 cm, consistent with our overall findings. Zhang Haili’s study (*n* = 414) [27] demonstrated that LR and LE were both safe and effective in the treatment of giant hemangiomas, which was also consistent with the overall conclusion of the present study. Notably, Professor Zhang reported greater intraoperative blood loss in the LE group compared with LR for giant hemangiomas (> 10 cm) located in the right hepatic lobe and caudate lobe. This finding contradicts the results of the right hepatic lobe subgroup analysis, in which the LE group was shown to be superior to the LR group in terms of blood loss, operation time, and occlusion time [15], similar results were observed in the relatively challenging right posterior lobe giant hemangiomas, but the advantage in reducing intraoperative blood loss was less pronounced. This discrepancy may stem from the technical complexities of right liver surgery. LR involves multiple steps, including the complete release of the perihepatic ligament, regional occlusion, and the delineation of the ischemic line, making it more complex and time-consuming. In contrast, LE is simpler and faster, involving tumor removal along the anatomical plane, which reduces intraoperative blood loss, portal occlusion time, and liver function damage.

For left hemi-liver hemangiomas, no significant differences were observed in operative time, blood loss, postoperative liver function markers (e.g., total bilirubin, albumin, prothrombin time), or other indices between LE and LR groups. However, LE group maximized preservation of normal liver tissue. The differences in outcomes between left and right hemi-livers may be attributed to anatomical factors. The left liver is more accessible and easier to be exposed. This simplifies both LR and LE, reducing operative complexity and minimizing differences between the two techniques. In contrast, right liver surgery requires extensive ligament release, regional occlusion, and ischemic line definition, increasing complexity, especially for LR group. Additionally, operative time, occlusion time, and blood loss significantly influence prognosis, with no significant differences observed for left hemi-hepatic hemangiomas.

Notably, the LR group for right hemi-liver hemangiomas required a higher proportion of autologous blood transfusions, likely due to increased intraoperative bleeding. This finding contrasts with the expectation that LR, with portal vein occlusion, would better control bleeding. The larger resection scope in LR may contribute to greater overall blood loss. To mitigate intraoperative bleeding risks during LE, we propose the following practical strategies based on our experience:1) In addition to routine imaging examinations, preoperative three-dimensional CT or MRI reconstruction to assess tumor size, location, adjacent structures, and vascular supply for precise surgical planning [6]; 2) Identifying an entry point with minimal liver tissue to facilitate tumor exposure and complete capsule peeling; 3) Protecting the tumor during enucleation, repeatedly confirming boundaries to maintain exposure tension and adhering to an “Easy-First” approach; 4) Using titanium clips for small tumor ruptures or suturing/ligation for larger ones, avoiding hemostatic materials for temporary bleeding control; 5) For tumors near the hepatic or portal vein, carefully delineating borders using blunt instruments (e.g., gauze) to avoid vein rupture, as hemangiomas typically do not adhere to surrounding structures. It is of paramount importance to refrain from any deliberate detachment or tearing of the tumor by means of a blind ultrasonic scalpel, as this could potentially result in the rupture of the vein. In the future, the safety and precision of laparoscopic LR and LE for giant hepatic hemangiomas may be further enhanced by advanced image-guided navigation and instrument technologies. Real-time navigation systems using magnetic microsensors have already been developed to improve spatial orientation and anatomical recognition during laparoscopic hepatectomy, potentially facilitating safer dissection near major vessels [28]. Complementary advances in force-sensing devices that quantify grip forces and associated histological damage in liver parenchyma may also help optimize tissue handling and reduce bleeding or ischemic injury during complex resections [29].

Postoperative bile leakage, a common and serious complication of liver surgery, is associated with intra-abdominal infections, impaired liver regeneration, and thromboembolic events, leading to prolonged hospital stays and increased readmission risks [30]. In this study, three cases of bile leakage occurred in right hemi-liver hemangiomas (one in LR, two in LE), with no significant difference between groups. The study suggests that intraoperative bile leakage was relatively common during laparoscopic liver resection and resulted in a higher incidence of postoperative bile leakage [31, 32], we should be aware of and appropriately manage intraoperative bile leakage to prevent postoperative bile leakage [33], we supported that intraoperative bile duct management follows strict operating specifications: fully exposing and clamping or ligating the target bile duct to ensure precise treatment, reducing postoperative complications. No other significant complications were observed, suggesting comparable safety and efficacy between LR and LE. Duration of follow-up, the extremely low recurrence risk of hepatic hemangioma following complete removal has been consistently demonstrated, irrespective of whether anatomical resection or enucleation is performed.

This study has several limitations that warrant cautious interpretation of the results. First, this is a retrospective study, which inevitably involves selection bias, significant selection bias arises from the non-randomized allocation of surgical approaches (LR vs. LE) based on patient preference, although counselling was standardized, potentially confounded by unmeasured factors such as education, risk aversion, tumor characteristics, or surgeon counseling. However, the choice of LE and LR driven by patient preference does not appear to compromise long-term lesional control in the current series, as no recurrence or residual hemangioma was observed in either group at a median follow-up of 24 months. Second, our study lacks analysis of giant hemangiomas located near major vessels or in the caudate lobe, where surgical challenges are greater. In the future, more detailed subgroup analyses are needed. Third, the retrospective, single-center design and limited sample size restrict generalizability, though consistent surgical techniques enhance internal validity. Fourth, the lack of a standardized decision algorithm and statistical adjustments (e.g., propensity score matching or multivariable regression) precludes robust causal inferences. Fifth, post-hepatectomy liver failure (PHLF) was not assessed using standardized international criteria (ISGLS definition) because total bilirubin and INR values on postoperative day 5 were not routinely available in all patients, potentially underestimating clinically significant liver dysfunction. Finally, multiple unadjusted comparisons for secondary outcomes increase the risk of Type I error, rendering findings exploratory. Future multicenter randomized controlled trials with predefined criteria and statistical controls are needed to validate these results and guide optimal management of giant hepatic hemangiomas.

## Conclusions

In summary, both LR and LE are safe and effective for treating giant hepatic hemangiomas. LE is recommended, particularly for right hemi-hepatic hemangiomas, due to shorter operative and occlusion times and reduced blood loss. For left hemi-hepatic hemangiomas, LE is preferred for its ability to preserve normal liver tissue. However, these findings should be interpreted with caution given the non-randomized, patient-preference study design, which introduces inherent selection bias. Future randomized controlled trials are essential to validate these conclusions and provide stronger evidence for surgical decision-making.

## Data Availability

The data that supports the findings of this study are included in this manuscript, and the original files are available from the corresponding author upon reasonable request.

## Abbreviations

HH: Hepatic Hemangioma
LR: Laparoscopic anatomic liver Resection
LE: Laparoscopic hemangioma Enucleation
ALT: Alanine Aminotransferase
AST: Aspartate Aminotransferase
TBIL: Total Bilirubin
PT: Prothrombin Time
PLT: Platelet
ALB: Albumin
BMI: Body Mass Index
ASA: American Society of Anesthesiologists
HGB: Hemoglobin
CRP: C-reactive Protein
POLS: Postoperative Length of Stay
KMS: Kasabach-Merritt Syndrome
PHLF: Post-Hepatectomy Liver Failure

## References

[1] Sadick M, Muller-Wille R, Wildgruber M, Wohlgemuth WA. Vascular anomalies (Part I): classification and diagnostics of vascular anomalies. Rofo. 2018;190(9):825–35. 29874693 10.1055/a-0620-8925

[2] Belghiti J, Cauchy F, Paradis V, Vilgrain V. Diagnosis and management of solid benign liver lesions. Nat Reviews Gastroenterol Hepatol. 2014;11(12):737–49. 10.1038/nrgastro.2014.151 25178878

[3] Yan C, Li BH, Sun XT, Yu DC. Laparoscopic hepatectomy is superior to open procedures for hepatic hemangioma. Hepatobiliary Pancreat Dis Int. 2021;20(2):142–6. 32980268 10.1016/j.hbpd.2020.09.001

[4] Jien H, Xiaohua L. Laparoscopic versus open surgery in the treatment of hepatic hemangioma: A meta-analysis. Med (Baltim). 2021;100(8):e24155. 10.1097/MD.0000000000024155 PMC7909164 33663045

[5] Jiang H, Chen Z, Prasoon P, Wu H, Zeng Y. Surgical management for giant liver hemangiomas greater than 20 cm in size. Gut Liver. 2011;5(2):228–33. 21814606 10.5009/gnl.2011.5.2.228 PMC3140671

[6] Yoshimizu C, Ariizumi S, Kogiso T, Sagawa T, Taniai M, Honda G, Egawa H, Tokushige K. Giant hepatic hemangioma causing prolonged fever and indicated for resection. Intern Med. 2022;61(12):1849–56. 34803101 10.2169/internalmedicine.8405-21 PMC9259820

[7] Adhikari DR, Thakur V, Telavane PP, Kulkarni R, Singh R, Joshi RM. Hypergiant hepatic hemangiomas: case series. Indian J Surg. 2015;77(Suppl 1):40–2. 25972639 10.1007/s12262-014-1104-8 PMC4425751

[8] Du X, Zheng K, Jiang L. Laparoscopic hepatectomy for giant hepatic hemangioma using the involved intrahepatic anatomic markers approach. J Gastrointest Surg. 2023;27(6):1290–1. 36877424 10.1007/s11605-023-05623-x

[9] Ghosh NK, Singh RR, Malage A, Sharma S, Kumar S, Singh A, Behari RK, Kumar A, Saxena A. Surgery for symptomatic hepatic hemangioma: resection vs. enucleation, an experience over two decades. Ann Hepatobiliary Pancreat Surg. 2023;27(3):258–63. 37127398 10.14701/ahbps.22-130 PMC10472124

[10] Jiang B, Shen ZC, Fang XS, Wang XM. Enucleation versus hepatectomy for hepatic hemangiomas: A meta-analysis. Front Surg. 2022;9:960768. 35965862 10.3389/fsurg.2022.960768 PMC9366102

[11] Zhang Z, Ji J, Qiu G, Hou Z, Mi S, Jin Z, Dai Y, Xie Q, Zeng Y, Huang J. Surgical indications for solid hepatic benign tumors: an updated literature review. Biosci Trends. 2023;17(5):325–34. 37599079 10.5582/bst.2023.01152

[12] Cheng WL, Qi YQ, Wang B, Tian L, Huang W, Chen Y. Enucleation versus hepatectomy for giant hepatic haemangiomas: a meta-analysis. Ann R Coll Surg Engl. 2017;99(3):237–41. 27869486 10.1308/rcsann.2016.0349 PMC5450283

[13] Wang Y, Ji W, Zhang X, Tan J. Laparoscopic liver resection and enucleation of liver hemangioma with selective hepatic vascular occlusion: technique and indications. J Laparoendosc Adv Surg Tech A. 2017;27(9):944–50. 27754755 10.1089/lap.2016.0432

[14] Dong J, Zhang M, Chen JQ, Ma F, Wang HH, Lv Y. Tumor size is not a criterion for resection during the management of giant hemangioma of the liver. Eur J Gastroenterol Hepatol. 2015;27(6):686–91. 25923944 10.1097/MEG.0000000000000344

[15] Li H, Duan X, Wu Z, Qin Y. Feasibility of laparoscopic enucleation for hemangioma in special hepatic segments. Front Surg. 2022;9:1111307. 36733682 10.3389/fsurg.2022.1111307 PMC9887023

[16] Dong Z, Fang K, Sui C, Guo J, Dai B, Geng L, Yang J. The surgical outcomes and risk factors of giant hepatic haemangiomas: a single centre experience. BMC Surg. 2022;22(1):278. 35843944 10.1186/s12893-022-01721-w PMC9290193

[17] Peng Z, Zhu ZR, He CY, Huang H. A meta-analysis: laparoscopic versus open liver resection for large hepatocellular carcinoma. Minim Invasive Ther Allied Technol. 2025;34(1):24–34. 38634257 10.1080/13645706.2024.2334762

[18] Toro A, Mahfouz AE, Ardiri A, Malaguarnera M, Malaguarnera G, Loria F, Bertino G, Di Carlo I. What is changing in indications and treatment of hepatic hemangiomas. A review. Ann Hepatol. 2014;13(4):327–39. 24927603

[19] Kaman L, Naik A, Savlania A, Raypattanaik N. Surgical management of giant hepatic Haemangioma - Need for redefining the nomenclature according to the size. Pol Przegl Chir. 2021;93(4):28–34. 34515653 10.5604/01.3001.0014.8105

[20] Tang T, Wang X, Mao Y, Li J, Wen T, Jia W, Chen Y, Peng T, Liu L, Fan R, et al. Real-world data on the clinicopathological traits and outcomes of hospitalized liver hemangioma patients: a multicenter study. Ann Transl Med. 2021;9(13):1067. 34422979 10.21037/atm-20-4684 PMC8339840

[21] Liu X, Yang Z, Tan H, Xu L, Sun Y, Si S, Liu L, Zhou W, Huang J. Giant liver hemangioma with adult Kasabach-Merritt syndrome: case report and literature review. Med (Baltim). 2017;96(31):e7688. 10.1097/MD.0000000000007688 PMC5626152 28767598

[22] Li L, Xu L, Wang P, Liu F, Wei Y, Xu M, Zhang M, Li B. Advantages of laparoscopic hepatic hemangioma surgery in quality of life: a prospective study. Surg Endosc. 2022;36(12):8967–74. 35701674 10.1007/s00464-022-09348-x

[23] Zhang W, Liu J, Zhang Z, Wang Y, Xiang S, Chen L, Zhu P, Zhang W, Shu C, Lau WY, et al. Perioperative outcomes of robot-assisted versus laparoscopic liver resection for cavernous hemangioma: a propensity score matching study. Surg Endosc. 2023;37(6):4505–16. 36810688 10.1007/s00464-022-09834-2 PMC10234931

[24] Xu L, Wu S, Kong J, Ke S, Yin T, Guo S, Ning C, Wang X, Li S, Ding J, et al. Thermal ablation of hepatic hemangioma: A multi-center experience with long-term outcomes. Eur J Radiol. 2023;164:110842. 37172442 10.1016/j.ejrad.2023.110842

[25] Torkian P, Li J, Kaufman JA, Jahangiri Y. Effectiveness of transarterial embolization in treatment of symptomatic hepatic hemangiomas: systematic review and Meta-analysis. Cardiovasc Intervent Radiol. 2021;44(1):80–91. 32808203 10.1007/s00270-020-02611-5

[26] Zhang W, Huang Z-Y, Ke C-S, Wu C, Zhang Z-W, Zhang B-X, et al. Surgical Treatment of Giant Liver Hemangioma Larger Than 10 cm: A Single Center's Experience With 86 Patients. Medicine. 2015;94(34):e1420. 10.1097/MD.0000000000001420 PMC4602926 26313792

[27] Zhang H, Xu H, Wen N, Li B, Chen K, Wei Y. Laparoscopic liver resection or enucleation for giant hepatic hemangioma: how to choose? Surg Endosc. 2024;38(6):3079–87. 38622227 10.1007/s00464-024-10820-z

[28] Igami T, Hayashi Y, Yokyama Y, Mori K, Ebata T. Development of real-time navigation system for laparoscopic hepatectomy using magnetic micro sensor. Minim Invasive Ther Allied Technol. 2024;33(3):129–39. 38265868 10.1080/13645706.2023.2301594

[29] Okuda Y, Nakai A, Sato T, Kurata M, Shimoyama I, Oda T, Ohkohci N. New device with force sensors for laparoscopic liver resection - investigation of grip force and histological damage. Minim Invasive Ther Allied Technol. 2022;31(1):28–33. 32468887 10.1080/13645706.2020.1755313

[30] Xue S, Wang H, Chen X, Zeng Y. Risk factors of postoperative bile leakage after liver resection: A systematic review and meta-analysis. Cancer Med. 2023;12(14):14922–36. 37326370 10.1002/cam4.6128 PMC10417307

[31] Epameinondas Dogeas STT, Geller DA. Does laparoscopic liver resection result in less leakage? J Am Coll Surg. 2022;234(2):112–4. 35213429 10.1097/XCS.0000000000000035

[32] Toro A. Is it better to intraoperatively diagnose biliary leakage after hepatic resection than to treat it postoperatively? J Am Coll Surg. 2022;235(3):567–8. 35709369 10.1097/XCS.0000000000000298

[33] Hayashi K, Abe Y, Shinoda M, Kitago M, Yagi H, Oshima G, Hori S, Wakabayashi T, Kitagawa Y. Clinical impact of intraoperative bile leakage during laparoscopic liver resection. Surg Endosc. 2020;35(8):4134–42. 32780232 10.1007/s00464-020-07880-2

